# Epidemiology of surgery in a protracted humanitarian setting: a 20-year retrospective study of Nyarugusu Refugee Camp, Kigoma, Western Tanzania

**DOI:** 10.1186/s12893-021-01365-2

**Published:** 2021-10-29

**Authors:** Sarah Rapaport, Hilary Ngude, Amber Lekey, Mohamed Abbas, Peter J. Winch, Kent Stevens, Zachary Obinna Enumah

**Affiliations:** 1 Global Surgery Initiative, Department of Surgery, Johns Hopkins Hospital, Baltimore, MD USA; 2 Tanzania Red Cross Society, Dar es Salaam, Tanzania; 3 Boston University School of Medicine, Boston, MA USA; 4 Department of International Health, Johns Hopkins School of Public Health, Baltimore, MD USA

**Keywords:** Global surgery, Refugee camp, Humanitarian setting, Global Health, Tanzania

## Abstract

**Background:**

There are 80 million forcibly displaced persons worldwide, 26.3 million of whom are refugees. Many refugees live in camps and have complex health needs, including a high burden of non-communicable disease. It is estimated that 3 million procedures are needed for refugees worldwide, yet very few studies exist on surgery in refugee camps, particularly protracted refugee settings. This study utilizes a 20-year dataset, the longest dataset of surgery in a refugee setting to be published to date, to assess surgical output in a setting of protracted displacement.

**Methods:**

A retrospective review of surgeries performed in Nyarugusu Camp was conducted using paper logbooks containing entries between November 2000 and September 2020 inclusive. Abstracted data were digitized into standard electronic form and included date, patient nationality, sex, age, indication, procedure performed, and anesthesia used. A second reviewer checked 10% of entries for accuracy. Entries illegible to both reviewers were excluded. Demographics, indication for surgery, procedures performed, and type of anesthesia were standardized for descriptive analysis, which was performed in STATA.

**Results:**

There were 10,799 operations performed over the 20-year period. Tanzanians underwent a quarter of the operations while refugees underwent the remaining 75%. Ninety percent of patients were female and 88% were 18 years of age or older. Caesarean sections were the most common performed procedure followed by herniorrhaphies, tubal ligations, exploratory laparotomies, hysterectomies, appendectomies, and repairs. The most common indications for laparotomy procedures were ectopic pregnancy, uterine rupture, and acute abdomen. Spinal anesthesia was the most common anesthesia type used. Although there was a consistent increase in procedural volume over the study period, this is largely explained by an increase in overall camp population and an increase in caesarean sections rather than increases in other, specific surgical procedures.

**Conclusion:**

There is significant surgical volume in Nyarugusu Camp, performed by staff physicians and visiting surgeons. Both refugees and the host population utilize these surgical services. This work provides context to the surgical training these settings require, but further study is needed to assess the burden of surgical disease and the extent to which it is met in this setting and others.

## Introduction

Worldwide, there are 80 million forcibly displaced persons (FDPs) including 26.3 million refugees, according to the United Nations High Commissioner for Refugees (UNHCR) [1]. 2.6 million refugees live in camps, which are temporary facilities intending to offer short term medical care, nutrition, shelter, and other basic emergency services [2]. Sixteen million are protracted refugees, defined as those who have lived in exile for at least 5 years and are unable to return home, but have not been granted permanent residence in another country [3].

FDPs and refugees have complex health needs, as they face the triple burden of non-communicable disease, infectious disease, and mental health needs [4]. Up to 3 million surgical procedures are needed for displaced persons worldwide according to a 2014 analysis, although this number is likely an underestimate as there were 59.5 million FDPs at the time of analysis, 20 million less than today [5]. Because 86% of displaced people are hosted in low- and middle-income countries (LMIC), refugees often present to care in settings with limited resources and constrained health systems [1]. Constrained health systems often lack safe, accessible, and affordable surgical care, with the Lancet Commission on Global Surgery reporting that 90% of the 5 billion people lacking surgical care worldwide reside in a LMIC [6]. Even though 28–32% of the global burden of disease is surgical in nature, research on surgery in refugee settings lags the demand for knowledge driven by the growing global surgery movement [7]. Despite a large increase in the global surgery literature with PubMed showing 570 cumulative articles published through 2005 and over 4000 through 2015, few studies exist on surgery in refugee settings [8]. Refugee camps have been relatively neglected from this field of literature and are even often included among the exclusion criteria [9].

The availability of data on surgical services in humanitarian settings is sparse, as care provision is often prioritized over research. The previous studies that do exist have focused on active conflict settings where the burden of trauma care is predominant [10–13]. Most that have assessed surgery in chronic humanitarian settings, such as the Mae La Camp on the Thai–Myanmar border, Dadaab Camp in Kenya, or Eastern Democratic Republic of Congo utilize datasets spanning 5 years or less [14–16]. The goal of this study is to describe patterns of surgery over a 20-year period from a setting of protracted displacement in Western Tanzania. To our knowledge, this is the largest retrospective registry of surgical output from a refugee setting.

## Methods

### Study setting and population

Nyarugusu Camp was created in 1996 for refugees fleeing civil wars in the Democratic Republic of Congo (DRC). It remains in existence today and is classified as a protracted refugee setting. Nyarugusu Camp is located in the Kigoma province in Western Tanzania, which has a long history of hosting refugees. Currently, Tanzania hosts over 286,000 refugees and asylum seekers with 85% residing in one of three camps: Nyarugusu as the largest, followed by Nduta and Mtendeli [17]. Nyarugusu Camp currently hosts about 133,000 refugees primarily from DRC and more recently Burundi, as political unrest in 2015 caused a dramatic increase in the number of Burundian refugees living in the camp [18]. As of July 2018, the camp population was 51% female and 55% of residents were under the age of 17, 42% were adults between the ages of 17–59, and 3% were aged 60 and over [19]. Nyarugusu Camp is run by the Tanzania Ministry of Home Affairs and the United Nations High Commissioner for Refugees (UNHCR), with additional operational and implementation support provided by a number of non-governmental (NGOs) and multilateral organizations including Oxfam, the UN World Food Program, UNICEF, and the International Rescue Committee to name a few. Chronic underfunding of this humanitarian health system, measured in April 2021 as operating at a deficit of $119,063,768 (only 8% of need is met) poses a challenge in this setting [20].

The Tanzanian Red Cross Society coordinates camp medical services, which includes supporting one main dispensary hospital, two health centers where patients can be admitted, and multiple health posts that serve both refugees and local Tanzanians. The total catchment population for the health centers and health posts is estimated to be approximately 200,000, and over 20,000 outpatients (refugees and Tanzanians) seek care at the hospital per month [21]. Although local Tanzanian district hospitals charge fees for both refugees and Tanzanians, services provided in the camp are free of cost for both refugees and Tanzanians [22]. If a case cannot be managed within the camp due to its complexity, refugees are referred to facilities outside of the camp according to UNHCR referral guidelines and local operating procedures [23].

The operating suite in Nyarugusu Camp consists of two major operating rooms and one minor operating room, and all performed procedures are recorded in paper logbooks that are maintained by camp staff. The major procedure operating room is reserved for procedures involving extensive resection (e.g. entering a body cavity or removing an organ). Surgeries are usually carried out by one of the medical doctors working with a humanitarian organization. In recent years, these are individuals who have completed medical school and an internship year. None have completed a formal residency in general surgery, but most have obtained some surgical training through apprenticeship. In more infrequent circumstances, visiting medical teams may also provide surgical care on repeated humanitarian mission trips (including authors KAS and ZOE). These trips have ranged from 1 to 6 weeks in duration. Within the timeframe of data collection, an earthquake damaged the former single operating room, which catalyzed the construction of a new building that houses the current two major operating rooms and one minor operating room.

### Data collection

A retrospective review of surgeries performed in the major operating room at Nyarugusu Camp was conducted using the paper surgery record logbooks containing entries between November 2000 and September 2020 inclusive. Abstracted data included the day, date, and time of surgery; patient nationality, sex and age; indication for procedure, procedure performed, anesthesia used, post-operative diagnosis, and Apgar score. Unfortunately, outcome data were only descriptive (“good”, “fair”) and inconsistently recorded, and therefore were not collected for this analysis. While mortality logbooks are maintained in this specific camp, there is no linked database to the operating room logbooks.

### Statistical analysis

Data were digitized from handwritten logbooks into standardized electronic form using Microsoft Excel and a second team member reviewed 10% of entries for accuracy. Each operation included between one and three procedures, one and three kinds of anesthesia, and some operations could have been performed on the same patient, as data was analyzed in a de-identified fashion for privacy reasons. Any entries that could not be read by either reviewer were marked as illegible. Demographics, procedure performed, indication for procedure, and type of anesthesia were standardized for analysis. Index operations that consisted of more than one procedure for a given patient (e.g., caesarean section plus bilateral tubal ligation or herniorrhaphy plus hydrocelectomy) were each coded to its respective operation as a binary variable. Descriptive analyses were performed for patient nationality, age, sex, procedure type, indication, and anesthesia used. Due to inconsistent reporting of time of day, post-operative diagnosis, and Apgar scores, these values were excluded from analysis. Of note, data were only available for November and December of 2000 and from January to September of 2020. Other years included data for all months of the calendar year. All analyses were performed using STATA statistical software (Version 16. StataCorp; College Station, TX).

### Ethics approval

This study was approved by both the Johns Hopkins Institutional Review Board and the Tanzanian Commission on Science and Technology (COSTECH). Informed consent was waived by the Johns Hopkins Institutional Review Board. All methods were conducted according to relevant guidelines and regulations.

## Results

### Demographics

A total of 10,799 unique operations were performed over the 20-year study period. After excluding entries for which the year variable was missing, there were a total of 10,780 operations (Table 1). Tanzanians underwent 25% of major operations (n = 2719). Fifty-five percent of refugees who had operations were from the Democratic Republic of Congo (DRC) (n = 5894), 16% were from Burundi (n = 1707) and 2% were from other nations (n = 158), which included Rwanda, Kenya, and those recorded in the logbook as “refugee.” An additional 3% (n = 302) were missing the nationality categorization, which are represented in the “missing” category. Ninety percent of the patients were female (n = 9722) and 88% were 18 years of age or older (n = 9539). Individuals aged between 18–29 years were the most represented age category (n = 6073; 56%) followed by those between 30–44 (n = 2670; 25%), pediatric patients between the ages of 0–17 (n = 1241; 12%), and patients over 60 (n = 224; 2%). An additional 2% (n = 225) were missing the age variable and are represented in the “missing” category.

**Table 1:** Demographics by 5-year time periods

	Overall 2000–2020	2000–2005	2006–2010	2011–2015	2016–2020
N	10,780^a^	1609	1812	3244	4115
Age (in years)					
Average (SD)	26.3 (10.6)	28.0 (13.6)	26.0 (10.6)	26.0 (10.3)	26.2 (9.5)
Under 18	1241 (11.5%)	245 (15.2%)	287 (15.8%)	424 (13.1%)	285 (6.9%)
Age 18 to 29	6073 (56.3%)	716 (44.5%)	919 (50.7%)	1779 (54.8%)	2659 (64.6%)
Age 30 to 44	2670 (24.8%)	382 (23.7%)	487 (26.9%)	859 (26.5%)	942 (22.9%)
Age 45 to 60	347 (3.2%)	84 (5.2%)	65 (3.6%)	111 (3.4%)	87 (2.1%)
Age 60+	224 (2.1%)	75 (4.7%)	29 (1.6%)	46 (1.4%)	74 (1.8%)
Missing	225 (2.1%)	107 (6.7%)	25 (1.4%)	25 (0.8%)	68 (1.7%)
Sex					
Male	992 (9.2%)	291 (18.1%)	173 (9.5%)	257 (7.9%)	271 (6.6%)
Female	9722 (90.2%)	1302 (80.9%)	1624 (89.6%)	2979 (91.8%)	3817 (92.8%)
Missing	66 (0.6%)	16 (1.0%)	15 (0.8%)	8 (0.2%)	27 (0.7%)
Nationality					
DRC	5894 (54.7%)	1228 (76.3%)	1337 (73.8%)	1868 (57.6%)	1461 (35.5%)
Burundi	1707 (15.8%)	2 (0.1%)	0 (0.0%)	267 (8.2%)	1438 (34.9%)
Tanzanian	2719 (25.2%)	286 (17.8%)	462 (25.5%)	981 (30.2%)	990 (24.1%)
Other	158 (1.5%)	7 (0.4%)	1 (0.1%)	84 (2.6%)	66 (1.6%)
Missing	302 (2.8%)	86 (5.3%)	12 (0.7%)	44 (1.4%)	160 (3.9%)

^a^Records that were missing a year variable were excluded (n = 19)

### Demographic changes over time

**Fig. 1:**
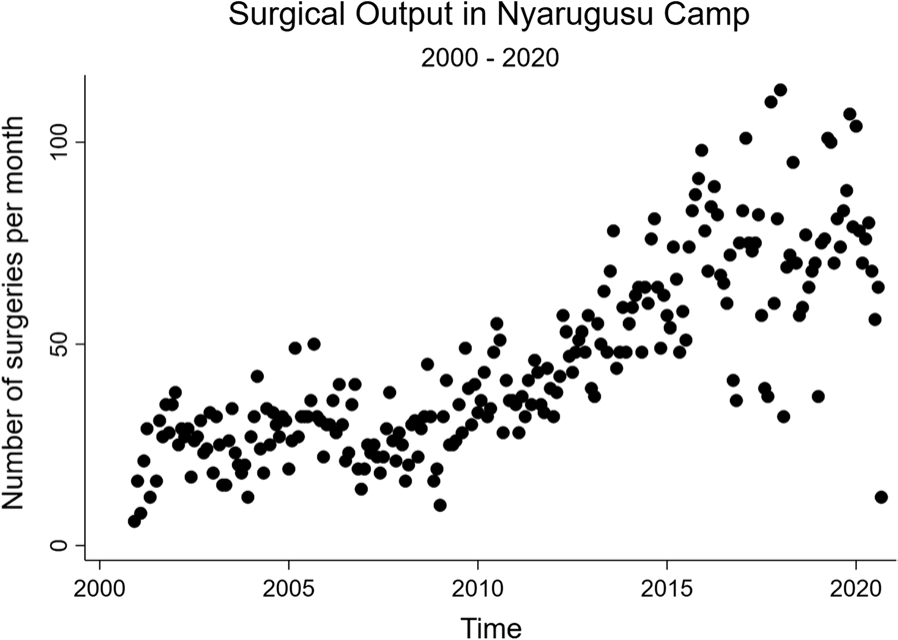
Total number of operations by month over time. *Of note, the year 2000 includes November and December only and the year 2020 includes January to September only

**Fig. 2:**
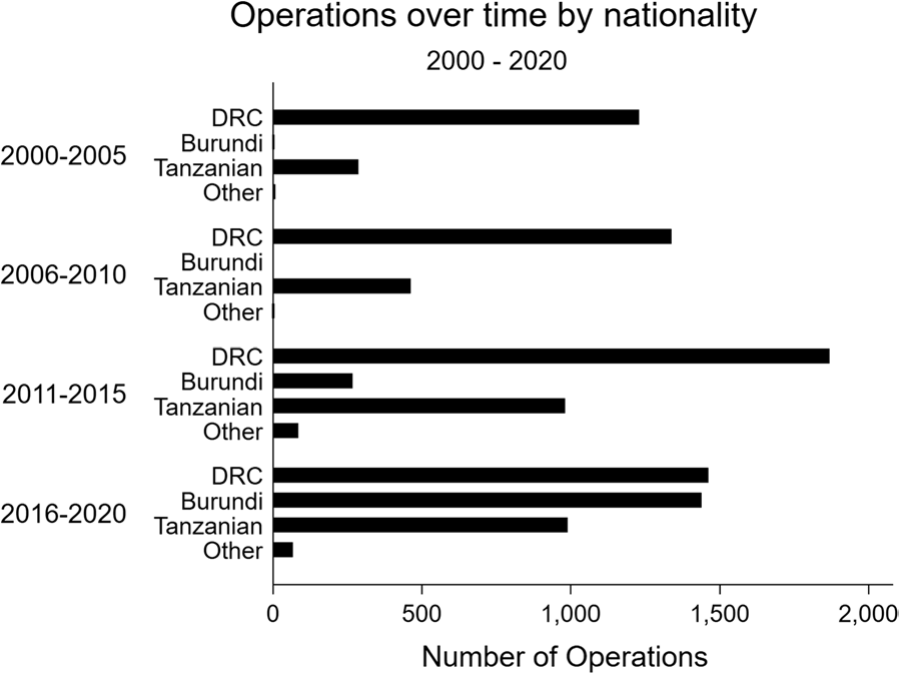
Operations by nationality over time

The volume of surgery increased over the 20-year study period (Table 2). When stratified into 5-year time periods, there was a steady increase in procedural output with 1609 procedures performed between November 2000–2005; 1812 performed between 2006–2010; 3244 performed between 2011–2015; and 4115 performed between 2016–September 2020 (Table 1; Fig. 1). While the study population composition of refugees (those from DRC, Burundi, and other) versus Tanzanians remained relatively constant over the study period, there was a drastic increase in the proportion of refugees from Burundi between 2016–2020 and a consequential decrease in the proportion of refugees from DRC over that same period (Table 1; Fig. 2).

**Table 2:** Operations by year and nationality

	Total	DRC	Burundi	Tanzanian	Other
N	10,492^a^	5898	1714	2722	158
Year					
2000	6 (0.1%)	0 (0.0%)	0 (0.0%)	0 (0.0%)	6 (3.8%)
2001	202 (1.9%)	166 (2.8%)	1 (0.1%)	35 (1.3%)	0 (0.0%)
2002	320 (3.0%)	266 (4.5%)	1 (0.1%)	53 (1.9%)	0 (0.0%)
2003	257 (2.4%)	206 (3.5%)	0 (0.0%)	51 (1.9%)	0 (0.0%)
2004	356 (3.4%)	270 (4.6%)	0 (0.0%)	85 (3.1%)	1 (0.6%)
2005	382 (3.6%)	320 (5.4%)	0 (0.0%)	62 (2.3%)	0 (0.0%)
2006	344 (3.3%)	276 (4.7%)	0 (0.0%)	68 (2.5%)	0 (0.0%)
2007	296 (2.8%)	235 (4.0%)	0 (0.0%)	61 (2.2%)	0 (0.0%)
2008	311 (3.0%)	230 (3.9%)	0 (0.0%)	81 (3.0%)	0 (0.0%)
2009	377 (3.6%)	258 (4.4%)	0 (0.0%)	119 (4.4%)	0 (0.0%)
2010	472 (4.5%)	338 (5.7%)	0 (0.0%)	133 (4.9%)	1 (0.6%)
2011	447 (4.3%)	345 (5.8%)	1 (0.1%)	101 (3.7%)	0 (0.0%)
2012	567 (5.4%)	379 (6.4%)	7 (0.4%)	175 (6.4%)	6 (3.8%)
2013	637 (6.1%)	405 (6.9%)	11 (0.6%)	220 (8.1%)	1 (0.6%)
2014	724 (6.9%)	414 (7.0%)	7 (0.4%)	267 (9.8%)	36 (22.8%)
2015	825 (7.9%)	325 (5.5%)	241 (14.1%)	218 (8.0%)	41 (25.9%)
2016	792 (7.5%)	290 (4.9%)	296 (17.3%)	170 (6.2%)	36 (22.8%)
2017	805 (7.7%)	293 (5.0%)	260 (15.2%)	224 (8.2%)	28 (17.7%)
2018	821 (7.8%)	282 (4.8%)	350 (20.4%)	188 (6.9%)	1 (0.6%)
2019	965 (9.2%)	388 (6.6%)	317 (18.5%)	259 (9.5%)	1 (0.6%)
2020	572 (5.5%)	208 (3.5%)	215 (12.5%)	149 (5.5%)	0 (0.0%)
Missing	14 (0.1%)	4 (0.1%)	7 (0.4%)	3 (0.1%)	0 (0.0%)

^a^Total 10,492 records given 307 records (2.8%) with missing data on nationality

### Procedures performed

**Fig. 3:**
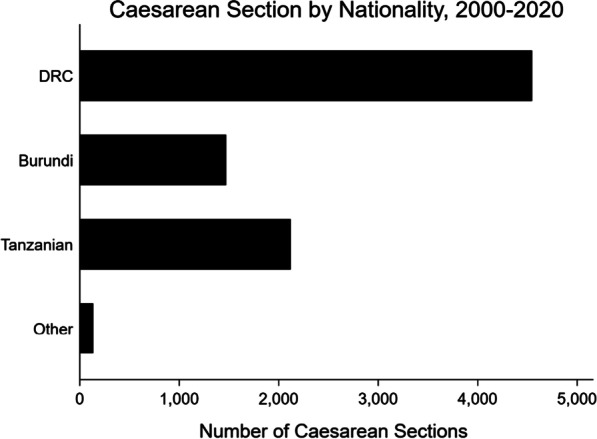
Caesarean sections by nationality over time

**Fig. 4:**
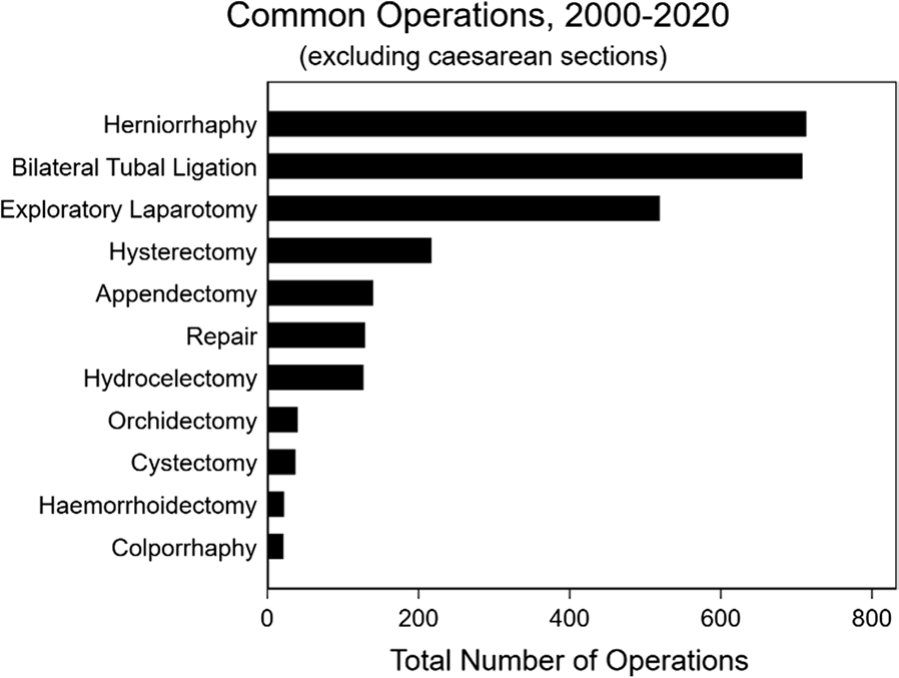
Common non-caesarean section surgeries

**Fig. 5:**
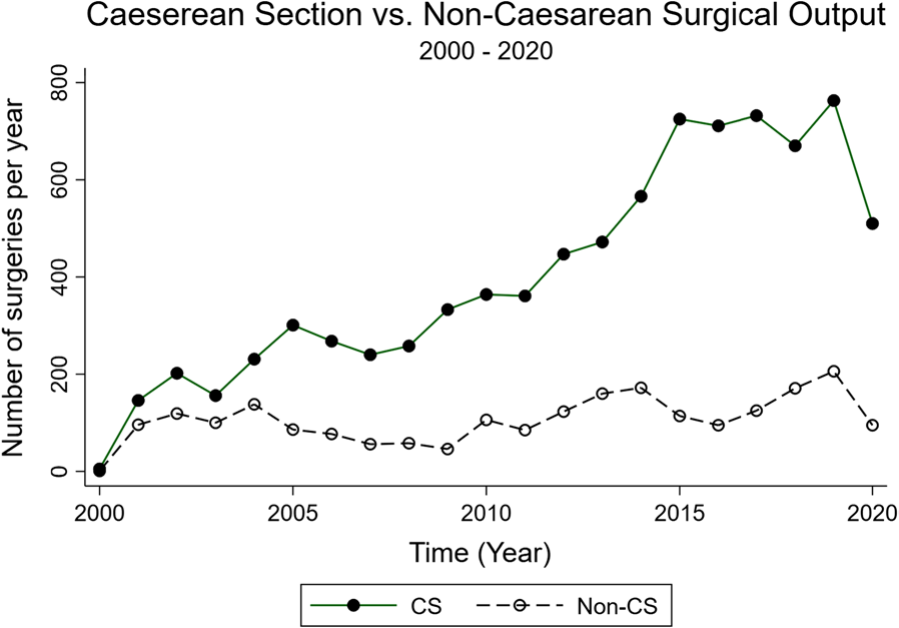
Total number of caesarean sections and non-caesarean section operations over time. *Non-caesarean section includes all observations in which a caesarean section was not performed. If, for example, a bilateral tubal ligation was performed concurrently with a caesarean section for the same patient/observation, this was not coded as non-CS for this figure

Caesarean sections were the most common procedure performed over the study period (n = 8461; 79%) (Table 3). Caesarean sections were most often performed on individuals from the DRC, followed by Tanzania, Burundi, and other nations (Fig. 3). Other common procedures include herniorrhaphies (n = 741, 7%), tubal ligations (n = 735; 7%), exploratory laparotomies (n = 555; 5%), hysterectomies (n = 220; 2%), appendectomies (n = 142; 1%), and repairs (n = 128; 1%) (Table 3; Fig. 4). Repair is a heterogeneous procedure code, and its indications span various organs and tissues in the body; example indications for a repair include perineal tear, cleft lip, abdominal perforation, cervical tear, and post-partum hemorrhage. Of note, procedures recorded as “repair” for a hernia were recorded as herniorrhaphy rather than repair. Although there was an increase in procedural volume over the study period, this is largely explained by an increase in total camp population and an increase in caesarean sections rather than increases in the performance of other kinds of surgical procedures (Fig. 5).

**Table 3:** Common operations over time

	Total	2000–2005	2006–2010	2011–2015	2016–2020
N	10,780	1609	1812	3244	4115
Caesarean section	8461 (78.5%)	1041 (64.7%)	1463 (80.7%)	2571 (79.3%)	3386 (82.3%)
Herniorrhaphy	741 (6.9%)	209 (13.0%)	124 (6.8%)	184 (5.7%)	224 (5.4%)
Bilateral tubal ligation	735 (6.8%)	106 (6.6%)	186 (10.3%)	273 (8.4%)	170 (4.1%)
Exploratory laparotomy	555 (5.1%)	131 (8.1%)	85 (4.7%)	182 (5.6%)	157 (3.8%)
Hysterectomy	220 (2.0%)	38 (2.4%)	32 (1.8%)	73 (2.3%)	77 (1.9%)
Appendectomy	142 (1.3%)	18 (1.1%)	55 (3.0%)	43 (1.3%)	26 (0.6%)
Repair	129 (1.2%)	3 (0.2%)	3 (0.2%)	47 (1.4%)	76 (1.8%)
Hydrocelectomy	129 (1.2%)	50 (3.1%)	22 (1.2%)	23 (0.7%)	34 (0.8%)
Orchidectomy	40 (0.4%)	2 (0.1%)	2 (0.1%)	22 (0.7%)	14 (0.3%)
Cystectomy	37 (0.3%)	7 (0.4%)	8 (0.4%)	11 (0.3%)	11 (0.3%)
Hemorrhoidectomy	22 (0.2%)	7 (0.4%)	1 (0.1%)	5 (0.2%)	9 (0.2%)
Colporrhaphy	21 (0.2%)	17 (1.1%)	0 (0.0%)	1 (0.0%)	3 (0.1%)
Missing	90 (0.8%)	28 (1.7%)	6 (0.3%)	19 (0.6%)	37 (0.9%)

### Indications for exploratory laparotomy

The most common indications for exploratory laparotomy were ectopic pregnancy (n = 84; 15%), uterine rupture (n = 76; 14%), acute abdomen (n = 74; 13%), ovarian cyst removal (n = 44; 8%), and intestinal obstruction (n = 27; 5%) (Table 4). Thirty-one entries (6%) that had the procedure of exploratory laparotomy were missing an indication.

**Table 4:** Common indications for exploratory laparotomy over time

	Total 2000–2020	2000–2005	2006–2010	2011–2015	2016–2020
N	555	131	85	182	157
Ectopic pregnancy	84 (15.1%)	31 (23.7%)	8 (9.4%)	23 (12.6%)	22 (14.0%)
Uterine rupture	76 (13.7%)	9 (6.9%)	7 (8.2%)	20 (11.0%)	40 (25.5%)
Acute abdomen	74 (13.3%)	9 (6.9%)	20 (23.5%)	34 (18.7%)	11 (7.0%)
Ovarian cyst	44 (7.9%)	11 (8.4%)	6 (7.1%)	15 (8.2%)	12 (7.6%)
Intestinal obstruction	27 (4.9%)	8 (6.1%)	10 (11.8%)	8 (4.4%)	1 (0.6%)
Missing	31 (5.6%)	11 (8.4%)	4 (4.7%)	7 (3.8%)	9 (5.7%)

### Anesthesia use

Spinal anesthesia was the most common anesthesia type used (n = 6963; 65%) followed by general (n = 3839; 36%), local (n = 95; 1%) and other (n = 61; 1%), which includes ketamine, atropine, and valium (Table 5). There were 229 entries (2%) that were missing an anesthesia categorization. There was considerable variation in the relative proportion of each anesthesia type between the 5-year time periods.

**Table 5:** Anesthesia over time

	Total	2000–2005	2006–2010	2011–2015	2016–2020
N	10,780	1609	1812	3244	4115
Spinal anesthesia	6963 (64.6%)	788 (49.0%)	1512 (83.4%)	1751 (54.0%)	2912 (70.8%)
General anesthesia	3839 (35.6%)	676 (42.0%)	427 (23.6%)	1545 (47.6%)	1191 (28.9%)
Local anesthesia	95 (0.9%)	62 (3.9%)	3 (0.2%)	3 (0.1%)	27 (0.7%)
Other anesthesia	61 (0.6%)	44 (2.7%)	3 (0.2%)	7 (0.2%)	7 (0.2%)
Missing	229 (2.1%)	102 (6.3%)	21 (1.2%)	21 (0.6%)	85 (2.1%)

## Discussion

We sought to explore patterns of surgical output in a protracted refugee setting in Western Tanzania. To our knowledge, this 20-year dataset is the largest analysis of surgical output in a humanitarian setting to be published to date. Our research not only illuminates the patterns of surgery in a protracted humanitarian setting, but also provides valuable quantitative data that can inform policy making, evidence-based program implementation, humanitarian surgical training, and global surgery capacity building efforts.

### Demographics

Demographic analysis revealed that an overwhelming majority of patients were female. Despite 51% of the camp population being female, this is likely because three of the top five most common operations were caesarean sections, tubal ligations, and hysterectomies. Additionally, there was a notable lack of pediatric surgery in the dataset, with patients under 18 representing only 11.5% of total operations despite this age group representing 55% of the camp population. Besides pediatric trauma, which would be represented in the laparotomy category and emergent surgery such as an appendectomy for appendicitis, other pediatric operations may be for congenital abnormalities and require a higher level of operative skill and specialization. These surgeries are not appropriate for management at this dispensary level hospital and are often referred to regional hospitals outside the camp and not recorded in the procedural logbooks. Approximately 25% of all surgeries performed in the camp were for Tanzanian patients, reflecting a large contribution of this camp-based hospital to treating the host population. While the percentage of Tanzanian patients remained relatively constant over the time period, between 2015 and 2020 there was a proportional decrease in patients from the DRC with a proportional increase in patients from Burundi. This largely reflects the shifting overall nationality demographics in the camp as individuals from Burundi arrived as a massive influx of refugees beginning in 2015. Increases in earlier years is likely the result of slow population increases in the camp as a whole.

### Characterizing chronic refugee surgery

Our research highlights that the landscape of surgery in this chronic refugee setting is more related to standard obstetric and general surgical needs rather than acute conflict related trauma care. This is consistent with findings from previous studies in humanitarian settings including those from Thailand, Kenya, and Eastern Democratic Republic of Congo, which found that this kind of disease burden significantly contributes to morbidity but that surgical intervention is cost effective and feasible [14–16]. Research in 1979–1980 from a chronic refugee setting of Cambodian refugees living in Khao I-Dang in Thailand estimated that non-war-related surgery outnumbered war related surgery three to one, emphasizing the need for non-war-related surgical care in these settings [24, 25]. Our study supports the findings of the previously mentioned ones with a high volume of obstetrical and general surgery procedures.

Our data also mirrors the literature on unmet surgical need in rural sub-Saharan Africa, where Grimes et al. notes caesarean sections and herniorrhaphies as the most common operations [26]. Grimes et al. also notes a lack of laparotomies, which is dissimilar to our finding of laparotomy as one of the most common procedures performed. Despite the lack of emergent, urgent, and elective procedure classification in our dataset, the indications of ectopic pregnancy, uterine rupture, acute abdomen, and intestinal obstruction in the exploratory laparotomy category represent a large amount of the urgent and emergent procedures. Our findings join the aforementioned studies in the notion that chronic refugee surgery differs from emergent refugee surgery, which is defined as care occurring immediately after a precipitating event [24]. Emergent refugee surgery is often trauma based and more closely related to conflict/war setting surgery, while chronic refugee surgery, defined by the literature and supported by our findings, is more related to the basic general surgical need found in underserved or rural settings. The lack of research on chronic refugee surgery is apparent and also documented by Kushner et al. and Broer et al. [24, 27]. While our research adds to the body of literature on surgery in the refugee camp context, our study does not include outcome data, which is generally lacking and needed in the humanitarian surgery space [28]. There is a great need to study surgery in chronic refugee populations worldwide to enable quality assurance, evidence based policy creation, and effective program implementation to better meet demonstrated need. Future studies should also include outcome data to better quantify the effectiveness of these efforts.

### Infrastructure limitations

Anesthesia availability, supported by the predominant use of spinal anesthesia, arose as a major limitation to the nature of procedures performed in this setting. Although challenging to ascertain how exactly this limitation affects procedural capacity, as caesarean sections utilizing spinal anesthesia are the primary procedure in this setting, this trend could show that anesthesia may influence which procedures can be performed in the camp hospital at which times, according to drug availability. An example of this may be the absence of thyroid surgery. This has implications for workforce training, as a provider must be adept at using a breadth of anesthesia options in a variety of conditions, including conditions and patients that may not be best suited to a particular anesthesia type or using anesthesia types different from what might be considered the standard approach in another context. Previous literature regarding anesthesia use in refugee camps is mixed. Data from Red Cross hospitals in Thailand, Lebanon, Pakistan, and Indonesia note that despite a strong preference for spinal and local anesthesia, general is the most commonly used anesthetic [29]. Research from an acute phase camp in Palestine notes that anesthesia was used based on availability and that once supplies diminished, more patients were required to use local or no anesthesia even when not ideal [30]. Our findings are consistent with the heterogeneity of anesthesia use. Although spinal is overall the most common anesthesia in our dataset, the frequency fluctuates between 5-year time periods suggesting anesthesia choice could be dependent on availability. The heterogeneity of this finding and its implications for limiting surgical capacity warrant further study in this setting and others.

The availability of trained surgeons is another limiting factor in humanitarian settings. Nyarugusu Camp has no formally trained general surgeons and surgical care is provided by either a visiting surgical team or Tanzanian general practitioners who have obtained some level of surgical skill. This ad hoc task shifting allows for some level of surgical care in this dispensary level hospital and aids both patients, for they are able to seek timely care at no charge, and Tanzanians, as referral hospitals can be spared the burden of care appropriately treated at dispensary level centers [31]. This care mirrors that provided in a district hospital. The human skill available likely biases the choice of procedures performed, potentially accounting for the majority of operations being caesarean sections. Task shifting in LMICs has been explored elsewhere in the literature. Previous research in Tanzania showed similar outcomes for procedures performed by surgeons and non-physician clinicians [32]. Previous research on task shifting in humanitarian settings has shown no difference in peri-operative mortality, which has prompted calls for the associate clinicians common among the global surgery workforce to be included in plans to build global surgery capacity, despite the recognition that more outcome data are needed [13, 33]. Our findings reflect those of the previous literature in that significant surgical volume can be supported by trained local clinical associates and medical doctors (not specialty trained surgeons) in conjunction with appropriate apprenticeship and mentoring. This explains how this camp, which has no formally trained surgeons, is able to perform a significant amount of surgery, ultimately providing care to a refugee and host population in need and preventing unnecessary referral care from straining national health systems. This has important implications for global surgery workforce training models, especially in humanitarian settings.

### Refugee surgery in the context of global surgery

Both our study and others show that humanitarian surgery services are utilized by host populations, particularly during times of health system stress, which reveals that these programs do not exist in a vacuum but rather contribute to larger national health systems [34]. Understudying refugee settings from the global surgery literature not only illuminates a false landscape of surgical needs and capacities worldwide, but also bypasses the valuable opportunity to improve humanitarian and host population surgical systems together.

## Limitations

Our study is not without limitations. Regarding the dataset itself, the logbooks are maintained by camp medical and administrative staff. Many of them are short term workers and there is heterogeneity and subjectivity in the recorded data. The lack of a standardized recording system, including regulation on how close to the time of a procedure an entry is made, introduces the potential limitation of accuracy of recorded data as it could lead to recall bias. Additionally, inconsistencies in the recorded variables, such as few entries containing post-operative diagnoses or Apgar scores, limited the variables that could be analyzed. This study assessed surgical output of the camp, which does not capture patients with surgical conditions treated at referral hospitals or those with surgical conditions either unaware of their surgical need or choosing not to seek treatment at the camp-based hospitals. As such, a household survey is needed to obtain population level data on the burden of surgical disease in the camp [35]. This has recently been employed in another refugee setting, but none have been conducted in sub-Saharan Africa [36]. Our group is currently conducting a household survey in Nyarugusu Camp. Finally, further delineating the landscape of surgical referral would also allow for a more complete picture of the burden and capacity of surgical disease in this refugee population.

## Conclusion

To our knowledge, this is the longest retrospective study of surgical output in a protracted refugee setting to date. Our findings reveal there is significant surgical volume in this dispensary hospital and a quarter of the patients utilizing these services are local Tanzanians. The most common operations are caesarean sections, herniorrhaphies, tubal ligations, and laparotomies and the most common indications for laparotomies are ectopic pregnancy, uterine rupture, and acute abdomen. The findings of this study can aid administrators in preparing and training the humanitarian workforce. Although this study contributes to the nascent body of literature on surgery in chronic refugee settings, there is a great need for further research related to operative and peri-operative outcomes, the unmet burden of surgical disease, and referral care relating to follow up and procedures deemed too specialized to be performed in the camp dispensary hospital. The growing number of refugees worldwide necessitates the inclusion of this population in global surgery advocacy and capacity building efforts. Further work in this setting and others is critical to formulating data driven policy and program implementation to benefit both the refugee and host populations that utilize camp-based services.

## Data Availability

Data used and analyzed in the current study are not publicly available due to privacy and personally identifiable health information. De-identified, aggregate data is available from corresponding author upon request.
